# Platelet Levels of Brain-Derived Neurotrophic Factor in Adults with Autism Spectrum Disorder: Is There a Specific Association with Autism Spectrum Psychopathology?

**DOI:** 10.3390/biomedicines12071529

**Published:** 2024-07-10

**Authors:** Barbara Carpita, Benedetta Nardi, Chiara Bonelli, Lavinia Pascariello, Gabriele Massimetti, Ivan Mirko Cremone, Stefano Pini, Lionella Palego, Laura Betti, Gino Giannaccini, Liliana Dell’Osso

**Affiliations:** 1 Department of Clinical and Experimental Medicine, University of Pisa, 56126 Pisa, Italy; barbara.carpita@unipi.it (B.C.); chiarabonelli.95@hotmail.it (C.B.); l.pascariello@studenti.unipi.it (L.P.); gabriele.massimetti@unipi.it (G.M.); ivan.cremone@gmail.com (I.M.C.); stefano.pini@unipi.it (S.P.); liliana.dellosso@unipi.it (L.D.); 2 Department of Pharmacy, University of Pisa, 56126 Pisa, Italy; lionella.palego@unipi.it (L.P.); laura.betti@unipi.it (L.B.); gino.giannaccini@unipi.it (G.G.)

**Keywords:** autism spectrum disorder, platelet BDNF, social anxiety disorder, suicidality, autistic traits, trauma related symptoms, BDNF

## Abstract

To date, although several studies have investigated the circulating levels of brain-derived neurotrophic factor (BDNF) in children with autism spectrum disorder (ASD), only a few authors have addressed their evaluation in adults. Furthermore, an important limitation of these studies lies in the fact that circulating BDNF is stored in platelets and released into the circulation when needed. To the best of our knowledge, a very limited number of studies have related peripheral BDNF values to platelet counts, and yet no study has evaluated intra-platelet BDNF levels in adults with ASD. In this framework, the aim of the present work is to pave the way in this field and evaluate platelet BNDF levels in adult ASD patients, as well as their correlation with autistic symptoms and related psychopathological dimensions. We recruited 22 ASD and 22 healthy controls, evaluated with the Adult autism subthreshold spectrum (AdAS Spectrum), the Social Anxiety Spectrum—self report (SHY-SR), the Trauma and loss spectrum—self report (TALS-SR), the Work and Social Adjustment Scale (WSAS), and the Mood Spectrum—self report for suicidality. Intra-platelet BDNF levels were also assessed. The results highlighted lower BDNF levels in the ASD group; moreover, AdAS Spectrum and WSAS total score as well as AdAS Spectrum Restricted interest and rumination, WSAS Private leisure activities, TALS-SR Arousal, and SHY-SR Childhood domains were significant negative predictors of platelet BDNF levels.

## 1. Introduction

Autism Spectrum Disorder (ASD) is a neurodevelopmental disorder characterized by a wide and heterogeneous range of manifestations. The core features of ASD are deficits in social interactions as well as in verbal and non-verbal communication, stereotyped and rigid behaviors, restricted interests, and altered reactivity to sensory stimuli [1]. This manifestation usually presents early in life and may or may not be accompanied by intellectual impairment and difficulties in language development [1]. Despite the fact that the majority of studies in the field of ASD have historically revolved around children, in recent years, the evaluation of ASD presentations in adulthood has gained growing attention. Many researchers particularly emphasized the significance of identifying milder forms of ASD in adult populations that do not involve intellectual disability, as these forms frequently go undiagnosed in childhood [2,3]. In fact, people with lighter forms of ASD frequently present for professional assessment following the emergence of other psychiatric conditions, the course of which is usually worsened by the co-occurring ASD [4,5]. Therefore, research in the field has recently emphasized the significance of assessing the presence of subthreshold autistic traits as well as milder forms of ASD. This is also because a growing body of research has linked these traits—even when they are below the threshold—to important clinical correlates, such as increased susceptibility to the emergence of mental illnesses, restricted ability to adjust to external stimuli and stressors, and suicidal ideation, as well as a greater chance of experiencing social phobic manifestations and developing trauma- and stress-related symptoms even from milder stressful life events [6,7,8,9]. In this framework, subthreshold autistic traits were originally investigated in family members of ASD subjects who were not clinically diagnosed with the disorder [10]. These relatives were frequently reported to exhibit personality traits that were reminiscent of their probands [11,12]. Recent reports have indicated that autistic traits are not exclusive to relatives of ASD probands but appear to be distributed across a continuum from the general to the clinical population [13,14,15,16,17,18], emphasizing the significance of examining the clinical and biological correlates of various autistic spectrum phenotypes. To date, most of the pathophysiological mechanisms related to ASD are yet unclear. A wide range of studies have suggested a major role both for genetic heritability [19,20,21] and environmental factors such as oxidative stress, maternal immunological dysregulation, and maternal diabetes and obesity [22,23]. In more recent years, many authors have focused on the investigation of possible biochemical mediators or markers of the disorder, mainly highlighting alterations in the immune and inflammatory response [24,25,26,27]. Furthermore, several studies have recently focused on the evaluation of circulating levels of brain-derived neurotrophic factor (BDNF) in autistic subjects [28,29,30]. Because synaptic growth, plasticity, and function depend on activity-dependent neuronal transmission, BDNF has become increasingly important due to its central role. Indeed, during postnatal brain development, BDNF plays specific functions in glutamatergic and GABAergic transmission, synaptic connections, synapse shape, neurotransmitter release, and synaptic plasticity.

Belonging to the neurotrophin family of secreted proteins, BDNF has a variety of roles in the growth, survival, and functionality of both central and peripheral neurons and is today recognized as the most prevalent and widely distributed neurotrophin in the central nervous system [31]. Pro-domain and mature BDNF proteins are released when the precursor protein proBDNF is broken down by either intracellular or extracellular proteases. BDNF was initially found in the brain, but it is now recognized to be present in the blood as well, where it is effectively kept in platelets. Platelets are the primary peripheral source of BDNF, with BDNF levels in them up to 100–1000 times higher than those in neurons. Like neurons, platelets mostly store BDNF in α-granules, which they release into the circulation when activated. It is interesting to note that while stimulation of platelets has been demonstrated to produce BDNF, the function of BDNF in platelets and the reasons behind its alterations are yet unknown. BDNF appears to modulate a wide variety of processes, including axon and dendritic guidance, growth, synapse formation, and the survival and differentiation of neurons. Moreover, it appears that BNDF aids in the differentiation and survival of dopaminergic neurons during neurodevelopment and controls synaptic plasticity during adulthood [31,32]. Many studies have evaluated circulating BDNF levels in both plasma and serum, assuming that they were correlated to BDNF levels in the brain, and have reported altered levels in several neurological and psychiatric disorders such as mood disorders, schizophrenia, and Alzheimer’s disease [33,34,35,36]. In particular, despite high heterogeneity, most of the studies that have investigated protein levels in ASD subjects report increased levels of BDNF in children on the spectrum [28,33,35,36]. In this framework, various effects of BDNF have been suggested to be linked to autistic symptomatology. For instance, changes in BDNF signaling in the central nervous system could account for the acceleration of brain growth and greater brain size as observed in children with ASD, as well as for the alterations in the connectivity, greater protein synthesis at synapses, greater dendritic spine density, enhanced synaptic plasticity. and enhanced sensory sensitivity [27,28,37]. However, such results have not always been confirmed, with some authors failing to report differences in peripheral BDNF levels in adults with ASD [38] and others describing lower BDNF levels in ASD children without intellectual impairment [27]. Some of the explanations proposed for those findings include the possibility of the presence of an age-mediated effect in BDNF levels for which elevated BDNF levels could be a compensating element in reaction to the brain’s delayed development [38] and the fact that lower levels of BDNF may be associated with a pro-inflammatory state and impaired neurogenesis in cortical regions [27].

Noticeably, an important limitation to the interpretation of these results is represented by the fact that peripheral BDNF is known to be stored within platelets and released when needed. Nonetheless, to our knowledge, to date, only one study has taken into account the potential confounding effects of platelet quantity [39] and specifically evaluated platelet BDNF in autistic subjects. In this context, the aim of our study was to evaluate intraplatelet levels of BDNF in a sample of adult autistic subjects without intellectual impairment and healthy controls. We also aimed to investigate possible correlations between platelet BDNF levels and a range of specific psychopathological dimensions often associated with ASD such as social phobic and trauma- and stress-related symptoms as well as social and work impairment.

## 2. Materials and Methods

### 2.1. Study Sample and Procedures

The total sample was made up of 44 subjects belonging to two diagnostic groups: 22 subjects with a diagnosis of ASD and 22 healthy controls (HCs). Participants belonging to the ASD group were recruited from in- and out-patients afferent to the Psychiatric Department of the Azienda Ospedaliera Universitaria Pisana (AOUP), University of Pisa, while the HCs were recruited on a voluntary basis. For both groups, exclusion criteria included age below 18 or over 65, the presence of an intellectual disability or language impairment that could prevent the completion of the questionnaires and/or the psychiatric evaluation, and a diagnosis of schizophrenia, substance use disorder, neurodegenerative disease, and any other relevant medical or neurological disorder. Moreover, exclusively for the HCs, another exclusion criterion regarded the presence of any psychiatric diagnosis according to the *Diagnostic and Statistical Manual of Mental Disorders—fifth edition*.

Each participant was assessed using a standardized clinical interview and psychometric tests. Each participant had a blood sample drawn in order to perform the biochemical evaluation. Before giving their written informed permission, all individuals were given thorough information about the study and given the chance to ask questions. The current study was conducted in compliance with the Helsinki Declaration, and the ethical committee in the area authorized all methods.

### 2.2. Psychometric Instruments

#### 2.2.1. Adult Autism Subthreshold Spectrum (AdAS Spectrum)

The AdAS Spectrum questionnaire is a self-report tool used to evaluate the spectrum of autistic manifestations in subjects without intellectual or linguistic impairments. It is made up of 160 dichotomous items divided into seven domains: *Childhood and Adolescence*, *Verbal communication*, *Non-verbal communication*, *Empathy*, *Inflexibility and Adherence to Routine*, *Restricted interests and rumination*, and *Hyper- and Hyporeactivity to Sensory Input*. The validation study revealed remarkable test–retest reliability (Kunder–Richiardson coefficient = 0.964, ICC = 0.976), great internal consistency, and convergent validity with other dimensional measures of autism [40,41].

#### 2.2.2. Work and Social Adjustment Scale (WSAS)

The WSAS is a questionnaire made of 5 items rated on a Likert scale ranging from 0 to 9, widely used to assess how symptoms affect one’s capacity for social and professional functioning, including the ability to work, manage one’s home, engage in private or social leisure activities, and establish and sustain close relationships. The instrument demonstrated strong internal consistency, with alpha coefficients ranging from 0.80 to 0.90 [42].

#### 2.2.3. Trauma and Loss Spectrum—Self Report (TALS-SR)

The TALS-SR is a self-report instrument, made up of 116 dichotomous items, organized into nine domains: *Loss events*, *Grief reactions*, *Potentially traumatic events*, *Reactions to losses or upsetting events*, *Re-experiencing*, *Avoidance and numbing*, *Arousal*, *Maladaptive coping,* and *Personal characteristics/risk factors*. The questionnaire is used to evaluate the lifetime incidence of different types of traumatic experiences in addition to personal traits, behaviors, and symptoms that could point to the presence of manifestations or risk factors for the development of a stress-related disorder. The questionnaire demonstrated good psychometric characteristics [43].

#### 2.2.4. Social Anxiety Spectrum—Self Report (SHY-SR)

The SHY-SR is a self-report questionnaire made up of 168 dichotomous items arranged into 5 domains: *Childhood and adolescence*, *Interpersonal sensitivity*, *Behavioral inhibition*, *Social situations*, and *Substance Abuse*. The questionnaire demonstrated good test–retest reliability, convergent validity with other dimensional measures of social anxiety, and significant internal consistency [44].

#### 2.2.5. Mood Spectrum—Self Report (MOODS-SR)

The MOODS-SR is a questionnaire designed to assess symptoms, behaviors, and lifestyle choices associated with different levels of mood dysregulation, including both severe and mild affective abnormalities. It is intended to be used in the evaluation of depression, mania, and hypomania. It consists of 160 items, divided into seven domains of which three assess manic aspects such as energy, mood, and cognition and three assess the same depressive characteristics. A further domain studies rhythmicity and vegetative processes, including eating, sleeping, and sexual activity.

The MOODS-SR was used in this study, as well as in earlier research, to measure suicidality or suicidal ideation and behaviors, as indicated by questions 102 through 107 [45,46,47].

### 2.3. Biochemical Evaluations

For each participant, after 12 h of fasting, 20 mL of peripheral venous blood was collected and stored in either tripotassium ethylenediaminetetraacetic acid (K_3_EDTA) to allow for the separation of the platelet-poor plasma (PPP) from the platelets, in a tube containing lithium–heparin for plasma separation, or in a tube without an anticoagulant and containing a coagulation activator for serum separation. Within 30 min of sample collection, the peripheral blood was centrifuged at low speed (150× *g*) for 15 min at room temperature to determine the precipitation of erythrocytes and leukocytes according to a density gradient. Subsequently, the supernatants of each subject were divided into two 15 mL Falcon tubes and the PRPs obtained were centrifuged at 1500× *g* for 15 min, allowing two Falcons to be obtained with the PPP and platelet precipitate (pellet) inside. At this point, the PPP was aliquoted into different test tubes so that each platelet pellet could be placed in the refrigerator at −80 °C while waiting to carry out the protein assay on the sample. The participant code, content, and initial volume of PRP from which the platelet sample was taken for subsequent intraplatelet BDNF calculations were indicated on each tube. All of the samples were maintained at −80 °C until the day of the assay. The intraplatelet content was determined using homogenization and fractionation techniques on the day of the BDNF assay, using a slightly modified procedure from that described in a previous study [48].

To measure the intraplatelet BDNF concentration in the samples, an Enzyme-Linked Immuno-Sorbent Assay (ELISA)—Sandwich type was used. The minimum concentration of mBDNF that the Biosensis kit used is able to detect is 7 pg/mL. In order to perform the competitive assay, the analyte as well as a first specific antibody were added to each well. Subsequently, for the detection reaction, a second biotinylated antibody linked to horseradish peroxidase (HRP) was added, followed by the HRP substrate, 3,3′,5,5′-tetramethylbenzidine (TMB). The final step of the assay was carried out using a multi-scan spectrophotometer to read the absorbance of the samples at 450 nm, as indicated in the kit instructions. The calibration curve was created using a 4-parameter nonlinear regression equation using Graph-Pad Prism Software (version 8.0, San Diego, CA, USA). The BDNF values were interpolated in logarithmic form and subsequently transformed into exponential form and multiplied by the dilution factors to obtain the final concentration of intra-platelet BDNF in ng/mL.

We followed Bradford’s method [49] to prevent biases related to individual variations in platelet count, adjusting the total protein values (mg/mL) that were achieved. The protein concentrations were expressed as ng/mg after normalization.

### 2.4. Statistical Analysis

Every statistical evaluation was performed with SPSS version 26.0.

Since our sample did not adhere to variance homoscedasticity or normality tests, we proceeded to use non-parametric techniques in the elaboration of our data.

In order to compare the socio-demographic variables, we used Chi-square and Mann–Whitney U-tests.

Scores obtained by the two groups in the different psychometric instruments employed as well as their BDNF levels were compared using the Mann–Whitney U-test.

Subsequently, in order to evaluate which psychometric measures were statistically predictive of platelet BDNF levels, a linear regression analysis was performed with platelet BDNF levels as the dependent variable and AdAS Spectrum, SHY-SR, TALS-SR total score, and suicidality as independent variables and another one with WSAS as an independent variable. Further linear regression analyses were performed using platelet BDNF levels as the dependent variable and AdAS Spectrum, WSAS, SHY-SR, and TALS-SR domain score as independent variables in order to investigate the presence of significant positive or negative predictors of platelet BDNF levels.

## 3. Results

The total sample was made up of 44 subjects divided into two diagnostic groups: 22 subjects with ASD and 22 HCs. The ASD group was made up of 15 (68.2%) males and 7 (31.8%) females with a mean age of 28.36 years (±6.97), while the HCs group was made up of 7 (31.8%) males and 15 (68.2%) females with a mean age of 33.91 years (±8.13). The groups significantly differed both in age and gender composition, with the ASD group being younger and with a higher prevalence of males (see Table 1).

As reported in Table 2, the results from the comparison between platelet BDNF levels between the two groups showed how ASD subjects had significantly lower levels of platelet BDNF compared to the HCs.

Similarly, the results from the comparison of the scores obtained in the different psychometric instruments between the ASD and HC groups showed how the ASD subjects scored significantly higher in all AdAS Spectrum, WSAS, SHY-SR, and TALS-SR domains and total score (see Table 3, Table 4, Table 5 and Table 6), as well as in the suicidality measures (see Table 7).

As reported in Table 8, the results from the linear regression analysis, including AdAS Spectrum, SHY-SR, TALS-SR and MOODS Suicidality score as independent variables, highlighted AdAS Spectrum as the only negative predictor of BDNF levels. A further regression analysis, performed with WSAS total score as the independent variable, highlighted a significant negative predictive effect on BDNF levels.

Another regression analysis performed with AdAS Spectrum domain scores as the dependent variable highlighted the AdAS Spectrum *Restricted interest and rumination* domain scores as a significant negative predictor of intraplatelet BDNF levels (see Table 9).

Lastly, the results from further linear regression analyses highlighted TALS-SR *Arousal* when including TALS-SR domains scores as the independent variable, SHY-SR *Childhood* when including SHY-SR total scores as the independent variable, and WSAS *Private leisure activities* when including WSAS items as the independent variable as significant negative predictors of platelet BDNF levels (see Table 10).

## 4. Discussion

According to our data, as expected, ASD subjects scored significantly higher in all AdAS Spectrum and WSAS domains and total score. From our results, ASD subjects also scored significantly higher in all SHY-SR domains and total scores. This evidence is in line with the recent literature that describes higher levels of social phobic traits in autistic subjects [50]. In fact, there are many similarities between ASD and social anxiety when it comes to social skills and engagement, and a number of reasons have been proposed as the causes of this convergence [51]. For instance, persistent difficulties in social settings may eventually cause social anxiety in certain people with ASD or autistic features [52]. Similarly, individuals with high-functioning ASD report low self-perceived social competence and heightened awareness of their communication difficulties; this may help explain why anxious symptoms appear in social settings [53]. Similarly, ASD subjects reported significantly higher scores in all TALS-SR domains and total scores, in accordance with the mounting data that suggest that not only ASD but also subthreshold autism features, may operate as risk factors for the development of trauma- and stress-related symptoms [5,54,55]. In particular, subjects with ASD are more vulnerable to bullying, rejection, and other socially stressful or even traumatic events due to their social problems and impaired socioemotional reciprocity, and this can lead to the development of trauma- and stress-related symptoms [54]. Lastly, ASD subjects scored significantly higher than HCs on suicidality measures. Still, this outcome is consistent with the wide body of literature that, over the past few years, has investigated the presence and correlation of suicidal thoughts and behaviors among autistic subjects [45,47,56,57,58]. Indeed, compared to their neuro-typical peers, subjects with autism are twice as likely to die by suicide and six times more likely to attempt suicide, sometimes at a far earlier age [59,60]. Furthermore, researchers have reported that at least one in six children with autism will consider suicide at some point in their childhood, even as young as six years old [61].

Interestingly, our results highlighted significantly lower levels of platelet BDNF in autistic subjects compared to HCs. BDNF is the most prevalent member of the neurotrophin family and is crucial for the growth and survival of neurons [62]. Mostly, it is widely recognized that BDNF has a significant role in the development of synaptic connections, including their creation, branching, and connectivity [63,64]. BDNF is distributed both inside the central nervous system and peripherally [65], and it has been demonstrated that circulating BDNF levels correspond to its levels in the brain [66,67]. Based on these premises, BDNF has been studied in many psychiatric disorders [68], and its peripheric concentrations in patients with ASD have been studied in various research, primarily finding greater levels of plasma BDNF, especially in children, with more controversial results among adults [28,37,69,70,71,72]. However, considering that peripheral BDNF is mostly stored in platelets and released after degranulation [73], a major limitation of these data arises from the fact that only a few studies have taken platelet counts into consideration when interpreting the results [39]. Although platelet-associated levels, receptors, and biomarkers and their implications have sometimes been studied in psychiatric disorders [74,75], to our knowledge, this is the first study that specifically aimed to evaluate platelet BNDF in adult ASD subjects. In this framework, the detection of low levels of platelet BDNF, hypothesizing greater platelet degranulation in autistic subjects [76], which on the one hand leads to an increase in circulating BDNF levels and on the other reduces intraplatelet levels, appears to be in line with the aforementioned data. Those results were confirmed by the regression analyses that highlighted AdAS spectrum total score as a negative predictor of platelet BDNF levels, implying that more severe pathology is related to greater alterations in BDNF levels and that, among the other dimensions included as independent variables (social anxiety, suicidality, and trauma- and stress-related symptoms) the autism spectrum dimension was indeed the one most linked to BDNF.

The finding of the SHY-SR *Childhood and Adolescence* domain as a negative predictor of BDNF levels can also be interpreted in light of the previous results. Indeed, the domain investigates relational deficits, difficulties in understanding and carrying out verbal and non-verbal communication, as well as difficulties in social interactions that manifest early in childhood, which are also typical of ASD [77,78,79,80]. Moreover, not only is social anxiety one of the most common co-occurrent disorders reported with ASD [81,82,83] but also, due to their similar presentation, it can sometimes be challenging to distinguish between the two disorders, specifically when they manifest in the earliest stages of life [84,85,86]. In this framework, many authors have hypothesized the presence of a common neurodevelopmental alteration in different psychiatric conditions, in particular for social anxiety, that could underpin the relationship between BDNF levels and early social phobic manifestations [13,14,49,87].

Interestingly, among autism spectrum dimensions, platelet BDNF levels were found to be negatively predicted by the AdAS Spectrum *Restricted interest and rumination* domain scores, investigating the tendency toward ruminative thinking, which increases the focus on feelings about problems rather than on problem-solving [40]. The most common definition of rumination as a psychiatric symptom is the act of persistently thinking about one’s own emotions and issues rather than thinking in terms of the specific content of one’s thoughts [88]. While ruminative thinking can be recognized as a central pillar of the autistic dimension, in recent years, many authors have focused on the study of this phenomenon not only in ASD [89] but also in a wide variety of mental disorders, suggesting its role as a trans-nosographic factor encompassing all psychopathology, with a detrimental role in the course and outcome [45,90,91,92]. Among the WSAS dimensions, the *Private leisure* activities domain score was a negative predictor of platelet BDNF levels. WSAS is a quick and accurate way to gauge functioning impairment, allowing one to evaluate how a person’s mental health issues affect their capacity to perform in a variety of situations and thus the overall severity of the psychiatric symptomatology [42]. Thus, the link between the WSAS score and BDNF levels reflects how more severe symptom pictures are associated with greater alterations [93,94,95] and is consistent with the aforementioned finding of the link between BDNF and ruminative thinking, as this in turn is frequently associated with greater severity of the psychiatric condition [45,90,91,92]. Moreover, the WSAS *Private leisure* activities domain explores the inability to experience pleasure in a variety of situations and therefore the state of anhedonia. Anhedonia, which is defined as a diminished desire for or ability to enjoy various activities, has been described in many psychiatric disorders such as major depression, schizophrenia, and autism [96,97,98,99,100] and is indicative of anomalies in the brain’s reward processing [101]. Interestingly, recent evidence has described how the brain’s reward system, which is essential for processing rewards, motivation, and pleasure-related actions, is greatly influenced by BDNF levels, which regulate brain plasticity in the mesolimbic dopamine system’s reward circuits [102,103,104]. Moreover, results from a recent study highlighted BDNF peripheric levels as possible independent predictors of consummatory anhedonia [102]. In this framework, it can be therefore hypothesized that changes in BDNF levels may cause a reduction in the sense of reward and ultimately lead to a state of anhedonia.

Interestingly, our results highlight TALS-SR *Arousal* domain score as a negative predictor of platelet BDNF levels. As previously stated, BDNF is present in many structures of the central nervous system and, in particular, the cortex, hypothalamus, hippocampus, and amygdala, which are involved in synaptic plasticity mechanisms that underpin learning and long-term memory [105]. In particular, between learning and memory processes, fear memories have a critical role in some mental illnesses, such as PTSD [106]. In fact, the primary feature of PTSD is the persistence of intensely painful memories associated with the incident, which is often accompanied by hyperarousal symptoms [1]. In this framework, altered peripheral BDNF levels have been reported in PTSD [107] and it has recently been described how BDNF may have a role in fear extinction and combat impaired extinction in anxiety disorders and PTSD [106]. Also, reduced peripheral BDNF has been reported in PTSD patients, particularly in the presence of chronic stress or long-lasting symptoms after exposure to the traumatic event as well as in relation to the type of encountered traumatism [108]. Despite the specific association between peripheral concentrations of BDNF and brain activity still being uncertain, some findings indicate that BDNF may play a role in the amygdala for the acquisition of fear conditioning and the consolidation of fear extinction; our results seem consistent with the evidence from BDNF studies, which indicate that altered BDNF levels are linked to extinction learning impairments [109,110].

Our results should be considered in light of some important limitations. First of all, our sample was small and with significant differences in sex and age, thus eventually affecting our results. Indeed, the ASD group was significantly younger and had a higher prevalence of male subjects compared to the HC group. The gender composition of our sample reflects the historical male predominance in the diagnosis of ASD, usually established at a 3:1 ratio in favor of males [111]. Moreover, we mostly used self-report questionnaires, which exposed our data to bias related to under- or over-estimation of symptoms. Also, the cross-sectional design of the study did not allow us to make inferences about causal or temporal relationships among the investigated variables. Lastly, it is noteworthy that platelets are essential suppliers of N-acetyl serotonin and serotonin [112]. ASD is classically associated with increased circulating serotonin levels [113], whilst levels of melatonin production across brain and systemic cells are decreased, including in platelets [114]. This is proposed to be mediated by an increase in microRNAs, including miR-451, that prevent the conversion of serotonin to N-acetyl serotonin and subsequently to melatonin [114]. Interestingly, N-acetyl serotonin is a BDNF mimic via the activation of the BDNF receptor, tyrosine kinase receptor (Trk)B [115], whilst N-acetyl serotonin can also induce BDNF, as shown in the hippocampal dentate gyrus [116]. How platelet BDNF interacts with the platelet tryptophan–serotonin-N-acetyl serotonin–melatonin pathway will be important to determine in future research, including how this is influenced by the gut microbiome [117].

In conclusion, despite these limitations, our study is not only one of the few taking into account platelets in the evaluation of BDNF levels but is possibly the only one to date to specifically address platelet BDNF levels in autistic subjects. Our results seem to point out the presence of significantly lower platelet BDNF levels in adults with ASD, and the possible association of BDNF reduction with specific psychopathological dimensions. In particular, our results also support the suggestion of a common neurodevelopmental alteration in different psychiatric conditions, and in particular for social anxiety, of a trans-nosographic role of rumination encompassing all psychopathology and of a newly suggested link between BDNF alterations and extinction learning impairments and the development of anhedonia, paving the way for further studies.

## Figures and Tables

**Table 1:** Age and sex in the overall sample and comparison between the diagnostic groups.

		ASD (n = 22) (Mean ± SD, Mean Rank)	HC (n = 22) (Mean ± SD, Mean Rank)	H	*p* *
**Age**		28.36 ± 6.97, 18.25	33.91 ± 8.13, 26.75	335.50	0.028 *
		**n (%)**	**n (%)**	**Chi-square**	***p***
**Sex**	**M**	15(68.2%)	7(31.8%)	5.82	0.016 *
	**F**	7(31.8%)	15(68.2%)		

*: statistically significant value (*p* < 0.05).

**Table 2:** Comparison of platelet BDNF levels among the groups.

	ASD (n = 22) (Mean ± SD, Mean Rank)	HC (n = 22) (Mean ± SD, Mean Rank)	H	*p* *
**Platelet BDNF ng/mg prot**	3.18 ± 1.25, 18.48	4.55 ± 2.46, 26.52	330.50	0.038 *

*: statistically significant value (*p* < 0.05).

**Table 3:** Comparison of AdAS Spectrum scores among the groups.

AdAS Spectrum	ASD (n = 22) (Mean ± SD, Mean Rank)	HC (n = 22) (Mean ± SD, Mean Rank)	H	*p* *
**Child./Adolesc.**	10.77 ± 3.69, 31.32	3.48 ± 2.11, 12.24	26.00	<0.001 *
**Verb. comm.**	10.27 ± 5.08, 31.61	1.57 ± 1.69, 11.93	19.50	<0.001 *
**Non-verb. comm.**	12.73 ± 4.50, 32.05	3.33 ± 2.22, 11.48	10.00	<0.001 *
**Empathy**	5.36 ± 2.87,31.20	0.86 ± 1.06, 12.36	28.50	<0.001 *
**Inflex. and routine**	21.73 ± 6.70, 32.18	5.33 ± 3.40, 11.33	7.00	<0.001 *
**Restrict. Interest and rum.**	13.27 ± 3.90, 32.39	3.05 ± 1.93, 11.12	2.50	<0.001 *
**Hyper-hyporeact.**	6.64 ± 3.37, 31.70	0.81 ± 1.17, 11.83	17.50	<0.001 *
**AdAS Spectr. total score**	80.77 ± 21.37, 33.07	20.91 ± 14.00, 11.93	9.50	<0.001 *

*: statistically significant value (*p* < 0.05).

**Table 4:** Comparison of WSAS scores among the groups.

WSAS	ASD (n = 22) (Mean ± SD, Mean Rank)	HC (n = 22) (Mean ± SD, Mean Rank)	H	*p* *
**Work**	5.39 ± 2.90, 28.69	0.45 ± 0.76, 11.23	14.50	<0.001 *
**Home management**	5.11 ± 2.56, 28.06	0.35 ± 0.67, 11.80	26.00	<0.001 *
**Social leisure activities**	5.17 ± 2.41, 28.67	0.25 ± 0.55, 11.25	15.00	<0.001 *
**Private leisure activities**	5.50 ± 2.75, 28.78	0.15 ± 0.37, 11.15	13.00	<0.001 *
**Close relationships**	4.06 ± 2.92, 27.56	0.10 ± 3.08, 12.25	35.00	<0.001 *
**WSAS total score**	25.22 ± 9.14, 28.78	1.30 ± 2.27, 11.15	13.00	<0.001 *

*: statistically significant value (*p* < 0.05).

**Table 5:** Comparison of SHY-SR scores among the groups.

SHY-SR	ASD (n = 22) (Mean ± SD, Mean Rank)	HC (n = 22) (Mean ± SD, Mean Rank)	H	*p* *
**Childhood**	6.50 ± 3.46, 28.40	2.77 ± 2.11, 15.23	82.00	<0.001 *
**Interpersonal sensitivity**	18.42 ± 5.27, 31.16	4.50 ± 4.80, 12.23	16.00	<0.001 *
**Behavioral inhibition**	10.68 ± 4.92, 31.18	1.45 ± 1.74, 12.20	15.50	<0.001 *
**Substance abuse**	1.65 ± 1.75, 26.23	0.54 ± 1.01, 17.20	125.50	0.010 *
**Social situations**	53.58 ± 20.33, 31.16	10.41 ± 11.39, 12.23	16.00	<0.001 *
**SHY-SR total score**	88.78 ± 29.64, 30.61	19.68 ± 18.02, 12.23	16.00	<0.001 *

*: statistically significant value (*p* < 0.05).

**Table 6:** Comparison of TALS-SR scores among the groups.

TALS-SR	ASD (n = 22) (Mean ± SD, Mean Rank)	HC (n = 22) (Mean ± SD, Mean Rank)	H	*p* *
**Loss**	4.20 ± 1.88, 26.05	3.00 ± 1.45, 17.36	129.00	0.020 *
**Grief Reactions**	13.10 ± 6.56, 27.80	6.50 ± 4.69, 15.77	94.00	0.001 *
**Potential Traumatic Events**	7.40 ± 3.42, 31.48	1.50 ± 1.40, 12.43	20.50	<0.001 *
**Reac. to Losses/Upset. Events**	9.48 ± 3.34, 28.45	4.23 ± 4.65, 14.57	67.50	<0.001 *
**Re-experiencing**	6.20 ± 2.09, 30.20	1.95 ± 2.46, 13.59	46.00	<0.001 *
**Avoidance/Numbing**	6.80 ± 3.14, 28.45	1.20 ± 2.89, 12.55	41.00	<0.001 *
**Maladaptive Coping**	3.35 ± 2.25, 28.55	0.57 ± 1.83, 13.81	59.00	<0.001 *
**Arousal**	2.85 ± 1.42, 30.45	0.45 ± 1.14, 13.36	41.00	<0.001 *
**Pers. charact.s/Risk Factors**	2.75 ± 1.65, 29.68	0.54 ± 0.96, 14.07	56.50	<0.001 *
**TALS-SR total score**	57.05 ± 17.85, 28.55	19.40 ± 16.01,11.88	27.50	<0.001 *

*: statistically significant value (*p* < 0.05).

**Table 7:** Comparison of suicidality scores among the groups.

	ASD (n = 22) (Mean ± SD, Mean Rank)	HC (n = 22) (Mean ± SD, Mean Rank)	H	*p* *
**Suicidality**	2.85 ± 2.16, 30.38	0.14 ± 0.64, 13.43	42.50	<0.001 *

*: statistically significant value (*p* < 0.05).

**Table 8:** Linear regression analyses with platelet BDNF levels as a dependent variable and AdAS Spectrum, SHY-SR, TALS-SR, and MOODS suicidality total score as independent variables (regression one) and WSAS total score (regression two) in the overall sample.

Linear Regression 1				
	b (SE)	BETA	t	*p*
**Constant**	4.77 (0.53)		9.059	<0.001 *
**AdAS Spectr. tot. score**	−0.018 (0.01)	−0.306	−2.083	0.043 *
R square = 0.094; Adjusted R square = 0.072				
**Linear Regression 2**				
**Constant**	4.37 (0.41)		10.716	<0.001 *
**WSAS tot. score**	−0.05 (0.02)	−0.336	−2.142	0.039 *
R square = 0.113; Adjusted R square = 0.088				

*: statistically significant value (*p* < 0.05).

**Table 9:** Linear regression analysis with AdAS Spectrum domain scores as the independent variable and platelet BDNF levels as the dependent variable.

	b (SE)	BETA	t	*p*
Constant	4.88 (0.51)		9519	0.001 *
**AdAS Spectrum—Restrict. int. and rum.**	−0.12 (0.50)	−0.354	−2428	0.020 *

R square = 0.126; adjusted R square = 0.104. *: statistically significant value (*p* < 0.05)

**Table 10:** Linear regression analyses with platelet BDNF levels as a dependent variable and TALS-SR domains (regression one), SHY-SR domains (regression two), TALS-SR domains (regression three) as independent variables.

	b (SE)	BETA	t	*p*
Constant	3.11 (0.88)		3.525	0.001 *
**TALS-SR Arousal**	−1.04 (0.49)	−0.888	−2.126	0.042 *
Constant	4.74 (0.53)		9.000	<0.001 *
**SHY-SR Childhood**	−0.19 (0.09)	−0.316	−2.053	0.047 *
Constant	4.47 (0.41)		11.006	<0.001 *
**WSAS Private leisure activities**	−0.23 (0.10)	−0.364	−2.347	0.025 *

*: statistically significant value (*p* < 0.05).

## Data Availability

All data generated or analyzed during this study are included in the published article.
